# Mental, social, and physical well-being in New Hampshire, Oregon, and Washington, 2010 Behavioral Risk Factor Surveillance System: implications for public health research and practice related to Healthy People 2020 foundation health measures on well-being

**DOI:** 10.1186/1478-7954-11-19

**Published:** 2013-09-24

**Authors:** Rosemarie Kobau, Carla Bann, Megan Lewis, Matthew M Zack, Angela M Boardman, Renee Boyd, Kim C Lim, Tommy Holder, Anastacia KL Hoff, Cecily Luncheon, William Thompson, Willi Horner-Johnson, Richard E Lucas

**Affiliations:** 1 Centers for Disease Control and Prevention, 4770 Buford Highway NE, MS-K78, Atlanta, GA 30341, USA; 2 RTI International, Research Triangle Park, 3040 Cornwallis Road, Durham, NC 27709-2194, USA; 3 Washington State Department of Health, Data Quality and Statistical Services (DQSS) Center for Health Statistics, P.O. Box 47814, Olympia, WA 98504-7814, USA; 4 Oregon Health Authority, Office of Disease Prevention and Epidemiology, Center for Health Statistics (Survey Unit), 800 NE Oregon St., Suite 225, Portland, OR 97232, USA; 5 New Hampshire Division of Public Health Services, Bureau of Public Health Statistics and Informatics, 29 Hazen Drive, Concord, NH 03301-6504, USA; 6 Institute on Development and Disability, Oregon Health & Science University, Portland, OR 97239, USA; 7 Department of Psychology, Michigan State University, East Lansing, MI 48824, USA

## Abstract

**Background:**

Well-being is now accepted as one of four cross-cutting measures in gauging progress for Healthy People 2020. This shift to population indicators of well-being redresses notions of health that have focused on absence of illness (negative health) as a primary or sufficient indicator of positive functioning. The purpose of this study was to estimate mental, social, and physical well-being in three US states using new measures piloted on the 2010 Behavioral Risk Factor Surveillance Survey System (BRFSS). Baseline estimates were provided for states overall, and within states for demographic subgroups, those with chronic health conditions or disabilities, and those with behavioral risk factors.

**Methods:**

Ten validated questions designed to assess mental (e.g., satisfaction with life, satisfaction with life domains, happiness), physical (e.g., satisfaction with energy level), and social dimensions (e.g., frequency of social support) of well-being were selected with state input for inclusion on BRFSS. 18,622 individuals responded to the BRFSS surveys administered by New Hampshire (N = 3,139), Oregon (N = 2,289), and Washington (N = 13,194). Multivariate adjusted proportions of positive responses to well-being items were examined.

**Results:**

After adjustment for confounders, about 67% of adults in these states had high levels of well-being, including >80% reporting experiencing happiness. Most adults were satisfied with their work, neighborhood, and education, but significant differences were seen in subgroups. Well-being differed by demographic characteristics such as marital status, health behaviors, chronic conditions, and disability status, with those who reported a disability and smokers consistently experiencing the worst well-being.

**Conclusions:**

Well-being is accepted as one of four cross-cutting measures in gauging progress for Healthy People 2020. Well-being differs by important sociodemographic factors and health conditions (e.g., age, employment, smoking, disability status). These findings provide baseline estimates for the three states to use in gauging improvements in well-being and can serve as a model for other state-level or national surveillance systems. These findings also assist states in identifying vulnerable subgroups who may benefit from potential interventions such as those in the National Prevention Strategy that focus on enhancing well-being where such disparities exist.

## 

Advances in the measurement of subjective well-being underlie the growing interest in monitoring this outcome in populations [1]. Well-being attempts to balance perspectives that have predominantly emphasized negative emotional states or outcomes as a way to understand functioning, or the primary use of economic indicators to measure population well-being [2]. The benefits of using well-being as a common framework for broad public policy have been described [1-5]. For the first time in its three-decade history, Healthy People 2020 (HP2020), a ten-year US federal initiative designed to engage multiple public and private sectors to improve population health, now supports monitoring population well-being as a cross-cutting measure to track progress in meeting HP2020 goals for preventing disease and injury, eliminating disparities, promoting healthy development, and improving quality of life [6]. This shift in how some health promotion goals will be measured now matches seminal declarations describing health as more than the absence of illness (“negative health”) [7-10], and is aligned with contemporary perspectives on positive health, inclusive of physical, mental, and social resources that actively promote well-being [11-15].

Well-being has been defined as evaluating life as satisfying and generally experiencing more positive states and emotions than negative ones [16,17]. Such evaluations may include meaning and purpose, affective reactions such as joy and sadness, and satisfaction with life as a whole as well as in domains such as work, family life, and housing [3]. These subjective evaluations and positive life orientations and experiences are related to a wide range of health outcomes including cardiovascular disease [18,19], immune functioning [20], and mortality [17,21]. Academic researchers have long studied well-being and its antecedents and consequences, but only recently have public health practitioners begun to focus on the importance of assessing well-being for resiliency, adaptation to illness, disease progression, and other health outcomes both within the United States [13,22-24] and internationally [1,7,25-27].

This focus is motivated by evidence showing that well-being is causally related to health and longevity [17]. Such assets or protective factors (e.g., positive affect, satisfaction, vitality) that compose well-being domains might serve to mediate protective physiological responses that are health enhancing (e.g., lower cortisol levels) or to more effectively moderate stressful responses (quell negative arousal), minimizing allostatic load (wear and tear on the body) [28-30]. Over time, these protective factors and processes may confer advantages such as greater resiliency associated with more successful age-related transitions over the life course [13,29].

Despite the burgeoning evidence linking well-being to health outcomes, including longevity [17], few surveillance systems in the US have collected extensive well-being data or examined variation by demographic factors, health behaviors, or conditions of interest to public health programs. Some surveillance systems have included single-item measures of global life satisfaction, happiness, and social or emotional support satisfaction [31]. These studies have related lower life satisfaction levels, operationalized with a single question, with greater prevalence of poor health, disability, smoking, obesity, and physical inactivity [31]. Moreover, the prevalence of smoking, obesity, physical inactivity, and heavy drinking increases as levels of social and emotional support decrease [32]. Other studies have revealed regional differences in well-being [33,34]. These difference may be associated with measurement issues (e.g., concept equivalence, response styles), cultural values (e.g., individualism vs. collectivism), socioeconomic factors (e.g., income levels, equality), or the interaction of these and other factors [35,36]. Widely used scales and items used in many countries and groups, such as the Satisfaction with Life Scale (SWLS), and overall happiness have been studied in relation to these cross-cultural issues [37-39]. The SWLS is one of the most extensively used and cross-culturally validated instruments in well-being research, demonstrating that asking people about what they think and how they feel about their lives offers valid information about an individual’s life circumstances and social context relative to other groups [38]. The SWLS also has shown acceptable convergent and discriminant validity with both subjective and objective well-being indicators [38]. Domain-specific life satisfaction items were developed for cross-cultural use, and have been shown to be robust measures [40]. Including multiple questions that tap into different well-being domains is useful for cross-cultural research [35]. Similarly, for US states, knowing whether certain demographic factors, health behaviors, or societal conditions are linked with well-being domains would provide a more detailed understanding of the experience of population well-being and could identify disparities in well-being among states, communities, and groups to guide local action [41]. This understanding could support future public health research and focus interventions and evaluations on enhancing population health.

Consistent with advances in the measurement of well-being, salutogenic approaches to health promotion [10,42], and in support of HP2020, the US Centers for Disease Control and Prevention (CDC) supported an initiative in 2007 to examine the feasibility of examining well-being beyond the use of single items for surveillance and health promotion [23]. For the first time, and with direct input on the selection of well-being questions from health departments in Oregon, New Hampshire, and Washington State, CDC included an expanded set of items from the SWLS, four domain-specific life satisfaction items selected by state health departments (e.g., satisfaction with present job, neighborhood, education, and energy level), and frequency of social/emotional support. The selected items were included on the 2010 Behavioral Risk Factor Surveillance System (BRFSS) as a pilot study. Measuring multiple domains that reflect social, mental, and physical functioning is consistent with public health definitions of well-being [8,23,43].

The present study extends previous well-being research by: (1) obtaining, for the first time, state-level baseline estimates of multiple well-being domains, including domain-specific life satisfaction, in representative populations; (2) assessing well-being as positive rather than negative functioning, using an expanded set of measures not previously used on BRFSS; (3) identifying population disparities in well-being within states to guide local prevention and promotion efforts; and (4) demonstrating the feasibility of using an expanded but brief set of measures that can be used by public health surveillance systems.

## Methods

### Survey

BRFSS is an ongoing, state-based, random-digit–dialed telephone survey of the civilian, non-institutionalized population aged 18 or older that tracks the prevalence of key health and safety-related behaviors and characteristics [44]. The questionnaire consists of (1) core questions asked in all 50 states, the District of Columbia, and US territories; (2) supplemental modules (i.e., a series of questions on specific health topics); and (3) state-added questions. Core questions are included in 22 sections, followed by supplemental modules and state-added questions. Each state decides which supplemental modules and state-added questions to include. Standardized questions on sociodemographic and behavioral characteristics as well as self-reported chronic diseases and activity limitations are included. The BRFSS survey is available at http://www.cdc.gov/brfss[44]. Data are weighted to reflect the age, sex, and racial/ethnic distribution of the state’s estimated population during the survey year [44].

### Measures

#### Mental well-being: Satisfaction with Life Scale

Mental well-being was assessed with a modified, validated version of the SWLS [37,45]. To account for the critical need for brevity on lengthy surveillance surveys or other program evaluation surveys concerned with respondent burden, CDC pilot tested a modified version of the SWLS (i.e., four items vs. five items, five-point vs. seven-point response scale, use of “my” [life] vs. “your” [life] in questions) for telephone surveillance purposes. The reliability remained acceptable (Cronbach alpha = 0.89 [CDC, unpublished data]), and use of a four-item scale is more feasible for surveillance purposes (Ed Diener, personal communication, May, 2009) [23]. Confirmatory factor analysis testing the modified SWLS with other gold standard measures supported its validity [45]. The four-item SWLS asked respondents to indicate how much they agree with the following statements on a scale from 1 (strongly agree) to 5 (strongly disagree): (1) “In most ways my life is close to ideal,” (2) “The conditions of my life are excellent,” (3) “I am satisfied with my life,” and (4) “So far I have gotten the important things I want in life.” Scores for the overall SWLS are calculated as the mean of the items.

#### Mental well-being: global life satisfaction and domain-specific life satisfaction

Domain-satisfaction is a valid dimension of well-being, serving as a key indicator for population well-being assessment [40]. Participating states recommended previously validated, specific life domains for inclusion [23]. States selected four of 13 possible domains previously examined in a nationally representative survey [23]. To maintain compbility with the global life satisfaction item, respondents were asked to rate how satisfied they were with the following components of their lives using a rating scale of 1 (very satisfied) to 4 (very dissatisfied): present job or work, neighborhood, education, and energy level.

#### Mental well-being: global happiness

BRFSS also includes a global life satisfaction question (“In general, how satisfied are you with your life?”) with response options from 1 (very satisfied) to 4 (very dissatisfied) [31,44]. The current study included a global happiness item as used on the 2001 National Health Interview Survey and other international surveys (“All things considered, would you say you are…”) with responses of 1 (very happy) to 5 (not happy at all) [39,46].

### Social well-being

The BRFSS social support item asks participants, “How often do you get the social and emotional support you need?” (this includes support from any source) [32]. Response options range from 1 (always) to 5 (never).

### Physical well-being: self-rated health

The BRFSS self-rated health question asks participants, “Would you say that in general your health is excellent, very good, good, fair or poor? Responses are rated from 1 (excellent) to 5 (poor). As part of this study, they were also asked about their vitality, an important physical domain indicator [47]. “In general, how satisfied are you with your energy level?” (Possible responses range from 1 (very satisfied) to 4 (very dissatisfied).

Standardized BRFSS variables for sociodemographic and behavioral characteristics (e.g., smoking, exercise) were used [44]. The physical well-being item, “self-rated health,” is the first question, in Section 1 (health status) of the BRFSS core survey, asked of all respondents. The BRFSS questions on social support and global life satisfaction were also part of the BRFSS core survey in 2010. These two questions were asked in Section 22 of the survey, as the last questions on the BRFSS core, preceding state-added modules. The question on satisfaction with social and emotional support was asked first, followed by the question on life satisfaction. The pilot well**-**being module, which included the global happiness item, the SWLS, domain-specific life satisfaction items, and the vitality item asked in this order, was the last module on BRFSS administered to respondents. The well-being module took an average of 105 seconds to administer.

### Statistical methods

Responses to well-being items were dichotomized into those indicating positive well-being (e.g., satisfied/very satisfied, agree/strongly agree) and those indicating negative well-being (e.g., dissatisfied/very dissatisfied, disagree/strongly disagree). For overall SWLS, scores of 4 or higher, corresponding to ratings of satisfied or very satisfied, were considered positive. Because other studies have found that sex, age, race/ethnicity, education, employment status, and related factors are correlated with well-being [23,36], we adjusted for these factors to avoid confounding. Adjusted percentages of positive responses to each item for demographic subgroups were estimated using logistic regression after controlling for state, gender, age, race/ethnicity, education, marital status, employment status, income, disability status, veteran status, chronic health condition (diabetes, heart attack, angina/coronary heart disease, stroke, or asthma), exercise, smoking, and obesity [48]. Adjusted percentages present estimates for all levels of an independent variable rather than for all but one level relative to a reference category (e.g., using white as a racial/ethnic reference group), and removes the difficulties of interpretation of measures of association [48]. We examined the percentages of the characteristics of respondents for each state and overall with respect to gender, age, race/ethnicity, education, marital status, employment status, income, disability status, veteran status, chronic health condition, physical activity, smoking status, and overweight/obesity. Non-overlapping 95% confidence intervals of adjusted percentages identify statistically significant differences in such percentages across subgroups, generally compble to a statistical significance level of 0.007, that partially adjust for multiple comparisons (similar to adjustment factors used when calculating p-values in multiple comparisons) [49]. Analyses were conducted using the SUDAAN statistical software program to account for the BRFSS’s complex survey design and sampling weights [50].

## Results

### Study participants

The current study included 18,622 adults who responded to the BRFSS surveys administered by New Hampshire (N = 3,139), Oregon (N = 2,289), and Washington (N = 13,194) (Table 1). Fifty-one percent are women; 53% are 45 years old or older; 83% are white, non-Hispanic; 69% have more than a high school education; 67% are currently married; 56%, are currently employed; 54% have annual household incomes of $50,000 or more; 27%, are disabled; 13% are military veterans; 27% have a chronic health condition; 82% had exercised in the past 30 days; 15% are current smokers; and 62% are overweight or obese. The three states did not differ in these characteristics except for greater percentages in New Hampshire than in Oregon of the employed and those with annual household incomes of $75,000 or more, and greater percentages in New Hampshire than in Washington of white, non-Hispanics.

**Table 1:** Demographic profile of respondents—Behavioral Risk Factor Surveillance System, New Hampshire, Oregon, and Washington, 2010

**Characteristic**	**All**	**New Hampshire**	**Oregon**	**Washington**
	**%**	**%**	**%**	**%**
Number	18,622	3,139	2,289	13,194
Gender				
Male	48.9	48.7	48.4	49.2
Female	51.1	51.3	51.6	50.8
Age				
18–24	11.1	9.3	11.6	11.2
25–34	17.3	13.9	16.3	18.1
35–44	18.5	21.2	16.4	18.8
45–54	19.9	21.4	19.7	19.8
55–64	16.2	16.0	17.5	15.7
65–74	9.5	10.0	10.1	9.2
75 or older	7.5	8.2	8.3	7.1
Race/ethnicity				
White	86.9	94.9	90.7	84.5
Black	1.5	0.8	1.0	1.8
Hispanic	6.1	2.0	4.2	7.4
Asian	3.4	1.1	1.7	4.4
American Indian/Pacific Islander	1.2	0.5	0.8	1.4
Other	0.8	0.8	1.6	0.6
Education				
Less than high school	6.1	4.3	5.4	6.7
High school graduate	24.9	26.7	27.5	23.7
More than high school	69.0	69.0	67.0	69.7
Marital status				
Married/living with partner	66.7	69.6	65.5	66.8
Divorced/septed	10.6	9.7	11.7	10.3
Widowed	5.1	5.6	5.5	4.9
Never married	17.6	15.0	17.4	18.1
Employment status				
Employed	56.3	62.8	49.8	57.8
Unemployed/unable to work	13.6	11.4	15.2	13.3
Retired	17.1	16.5	19.1	16.5
Homemaker/student	13.0	9.4	15.9	12.5
Income				
< $15,000	6.3	5.0	7.5	6.1
$15,000–$19,999	5.2	5.0	6.7	4.7
$20,000–$24,999	9.1	6.9	10.3	9.0
$25,000–$34,999	10.1	9.0	9.6	10.4
$35,000–$49,999	14.8	15.0	16.0	14.4
$50,000–$74,999	19.0	18.3	20.0	18.7
≥ $75,000	35.4	40.7	29.9	36.6
Disabled	27.2	22.3	29.6	27.0
Military veteran	13.0	14.3	13.3	12.7
Chronic health condition	26.5	25.7	28.6	25.9
Exercise in past 30 days	82.0	79.2	82.5	82.2
Current smoker	15.2	16.0	15.0	15.2
Overweight/obese	61.7	61.9	61.6	61.7

Note: Chronic health conditions include self-reported doctor-diagnosed diabetes, heart attack, angina/coronary heart disease, stroke, and asthma. Percentages are weighted.

On average, less than 2% of responses to the mental, social, and physical well-being items were classified as “don’t know/refused”.

### Mental well-being

#### Life satisfaction

**Figure 1:**
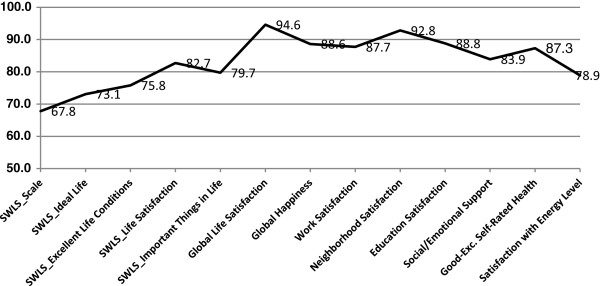
**Overall percent agreement with well-being domains, 2010 BRFSS pilot study, NH, OR, WA.** SWLS = Satisfaction with Life Scale [37]. For overall SWLS (“SWLS_Scale”), scores of 4 or higher, corresponding to ratings of satisfied or very satisfied, were considered positive. SWLS_Scale is based on positive responses to the four items used in this study: “In most ways, my life is close to ideal; The conditions of my life are excellent; I am satisfied with my life; So far, I have gotten the important things I want in life”.

Based on the modified SWLS, after controlling for state, demographic and health characteristics, 68% of respondents reported positive life satisfaction (Table 2). At the item level, 73% reported that their lives were close to ideal, 76% thought the conditions of their lives were excellent, 83% reported being satisfied with their lives, and 80% felt they had gotten the important things in life (Figure 1). Demographic differences in life satisfaction were generally similar across individual items and the overall scale; findings for the overall scale follow.

**Table 2:** Adjusted proportions of agreement with Satisfaction with Life Scale items and overall Satisfaction with Life Scale by demographic characteristics, chronic health condition status, select behavioral risk factors, and state—Behavioral Risk Factor Surveillance System, New Hampshire, Oregon, and Washington, 2010

**Characteristic**	**Satisfaction with Life Scale individual items**				**Satisfaction with life**
	**“In most ways my life is close to ideal”**	**“The conditions of my life are excellent”**	**“I am satisfied with my life”**	**“So far I have gotten the important things I want in life”**	**Scale (Overall)**
	**Percent (95% CI)**	**Percent (95% CI)**	**Percent (95% CI)**	**Percent (95% CI)**	**Percent (95% CI)**
N	18,339	18,391	18,447	18,394	18,527
Overall	73.1 (71.9, 74.2)	75.8 (74.7, 76.8)	82.7 (81.7, 83.6)	79.7 (78.6, 80.8)	67.8 (66.6, 69.0)
Gender					
Male	72.7 (70.9, 74.4)	75.2 (73.4, 76.8)	82.1 (80.5, 83.6)	77.5 (75.8, 79.2)	66.1 (64.2, 67.9)
Female	73.5 (71.9, 75.0)	76.4 (74.9, 77.8)	83.2 (81.8, 84.5)	81.8 (80.4, 83.1)	69.5 (67.8, 71.1)
Age					
18–24	75.1 (69.1, 80.3)	79.0 (73.5, 83.7)	85.8 (81.0, 89.6)	79.2 (74.0, 83.7)	71.4 (65.1, 76.9)
25–34	72.1 (68.6, 75.4)	75.1 (71.7, 78.2)	81.6 (78.3, 84.5)	76.9 (73.4, 79.9)	66.5 (62.8, 69.9)
35–44	72.9 (70.3, 75.3)	74.5 (72.0, 76.8)	81.1 (78.8, 83.2)	78.6 (76.2, 80.8)	67.0 (64.4, 69.6)
45–54	69.1 (66.9, 71.2)	71.1 (69.0, 73.2)	79.0 (77.0, 80.8)	78.3 (76.3, 80.2)	62.7 (60.5, 64.9)
55–64	73.3 (71.5, 75.1)	76.2 (74.4, 77.9)	82.7 (81.1, 84.2)	81.4 (79.8, 83.0)	68.0 (66.0, 69.9)
65–74	77.3 (74.8, 79.6)	80.2 (77.9, 82.3)	86.5 (84.5, 88.3)	84.8 (82.4, 86.8)	73.5 (71.0, 75.9)
75 or older	77.9 (74.7, 80.8)	80.8 (77.9, 83.4)	88.3 (85.9, 90.3)	84.6 (81.7, 87.1)	73.5 (70.1, 76.5)
Race/ethnicity					
White	72.9 (71.6, 74.1)	75.5 (74.3, 76.7)	82.4 (81.4, 83.5)	80.1 (78.9, 81.2)	67.6 (66.3, 68.9)
Black	65.1 (55.6, 73.6)	76.8 (68.0, 83.7)	79.9 (71.3, 86.4)	71.6 (62.9, 79.0)	65.0 (55.8, 73.2)
Hispanic	79.0 (73.8, 83.5)	78.9 (73.9, 83.2)	88.3 (84.2, 91.5)	82.4 (77.5, 86.4)	73.2 (67.7, 78.1)
Asian/Pacific Islander	71.3 (62.8, 78.5)	75.8 (67.1, 82.8)	79.8 (70.6, 86.7)	68.4 (60.7, 75.2)	61.5 (53.4, 69.0)
American Indian/Alaskan Native	71.5 (59.2, 81.3)	73.8 (62.5, 82.7)	82.3 (72.4, 89.1)	80.8 (71.2, 87.8)	68.3 (56.4, 78.2)
Other	76.8 (65.5, 85.3)	79.1 (69.0, 86.6)	81.4 (71.4, 88.4)	81.6 (71.3, 88.7)	75.0 (63.7, 83.7)
Education					
Less than high school	70.2 (64.5, 75.3)	74.0 (69.0, 78.5)	82.0 (77.4, 85.8)	80.7 (76.1, 84.6)	65.3 (59.5, 70.7)
High school graduate	73.2 (71.0, 75.4)	74.8 (72.6, 76.8)	83.7 (81.9, 85.4)	79.1 (77.0, 81.1)	67.4 (65.0, 69.7)
More than high school*	73.3 (71.9, 74.6)	76.3 (75.0, 77.6)	82.3 (81.0, 83.5)	79.9 (78.5, 81.1)	68.1 (66.7, 69.5)
Marital status					
Married/living with partner	77.2 (75.9, 78.5)	79.0 (77.7, 80.3)	85.9 (84.8, 86.9)	84.5 (83.3, 85.7)	72.9 (71.5, 74.3)
Divorced/septed	64.6 (61.4, 67.7)	67.4 (64.4, 70.3)	74.6 (71.5, 77.4)	71.2 (68.0, 74.3)	56.7 (53.4, 59.9)
Widowed	70.0 (66.3, 73.4)	72.1 (68.5, 75.4)	79.2 (75.6, 82.4)	76.3 (72.5, 79.7)	62.8 (59.0, 66.4)
Never married	63.2 (59.1, 67.2)	70.4 (66.6, 73.9)	77.2 (73.9, 80.1)	68.8 (65.0, 72.4)	55.1 (50.7, 59.5)
Employment status					
Employed	73.2 (71.6, 74.7)	76.5 (75.0, 77.9)	83.8 (82.5, 85.0)	79.9 (78.5, 81.3)	67.7 (66.1, 69.3)
Unemployed/unable to work	65.5 (61.7, 69.0)	66.8 (63.1, 70.3)	74.2 (70.8, 77.2)	73.2 (69.8, 76.4)	58.1 (54.2, 62.0)
Retired	78.4 (76.1, 80.6)	80.2 (77.9, 82.3)	86.3 (84.2, 88.1)	85.0 (82.8, 87.0)	74.4 (72.0, 76.7)
Homemaker/student	74.1 (70.2, 77.7)	77.1 (73.5, 80.4)	84.0 (80.4, 87.0)	80.4 (76.6, 83.7)	69.3 (65.2, 73.2)
Income					
< $15,000	68.7 (63.6, 73.4)	66.6 (61.4, 71.4)	79.7 (75.9, 83.0)	72.2 (67.4, 76.6)	60.9 (55.4, 66.1)
$15,000–$19,999	66.5 (60.8, 71.7)	63.9 (57.7, 69.6)	77.7 (72.8, 82.0)	71.6 (66.3, 76.3)	57.1 (50.9, 63.0)
$20,000–$24,999	63.4 (58.8, 67.8)	66.4 (61.9, 70.6)	76.3 (72.1, 80.0)	73.7 (69.5, 77.5)	58.3 (53.6, 62.9)
$25,000–$34,999	65.6 (61.9, 69.1)	68.0 (64.5, 71.4)	77.3 (73.9, 80.4)	75.4 (71.7, 78.7)	60.6 (57.0, 64.2)
$35,000–$49,999	71.1 (68.4, 73.7)	74.8 (72.3, 77.2)	81.8 (79.4, 83.9)	75.5 (72.6, 78.2)	64.1 (61.2, 66.8)
$50,000–$74,999	73.7 (71.0, 76.2)	77.3 (74.9, 79.6)	83.4 (81.2, 85.4)	82.2 (79.7, 84.4)	68.6 (65.9, 71.2)
≥ $75,000	80.4 (78.3, 82.3)	84.2 (82.3, 85.9)	87.9 (86.1, 89.4)	86.6 (84.7, 88.2)	76.3 (74.1, 78.3)
Disability status					
Yes	62.9 (60.5, 65.3)	65.4 (63.0, 67.7)	74.0 (71.6, 76.2)	74.2 (72.0, 76.4)	57.2 (54.8, 59.6)
No	76.9 (75.6, 78.2)	79.8 (78.6, 81.0)	86.2 (85.1, 87.2)	81.9 (80.6, 83.0)	71.7 (70.3, 73.0)
Veteran					
Yes	71.5 (68.4, 74.5)	74.7 (71.6, 77.6)	81.7 (78.7, 84.4)	78.7 (75.9, 81.2)	65.2 (62.1, 68.3)
No	73.3 (72.1, 74.5)	75.9 (74.8, 77.1)	82.8 (81.7, 83.8)	79.9 (78.7, 81.0)	68.2 (66.9, 69.4)
Chronic health condition					
Yes	73.4 (71.2, 75.4)	75.8 (73.8, 77.6)	83.0 (81.3, 84.6)	79.4 (77.5, 81.3)	67.9 (65.6, 70.0)
No	73.0 (71.6, 74.3)	75.8 (74.5, 77.0)	82.5 (81.3, 83.6)	79.8 (78.5, 81.1)	67.8 (66.4, 69.1)
Exercise in past 30 days					
Yes	73.7 (72.4, 74.9)	76.2 (74.9, 77.3)	83.2 (82.1, 84.3)	79.9 (78.7, 81.1)	68.2 (66.9, 69.5)
No	70.5 (68, 73.0)	74.2 (71.9, 76.4)	80.4 (78.3, 82.4)	79.0 (76.7, 81.1)	66.0 (63.3, 68.5)
Current smoker					
Yes	63 (59.7, 66.2)	67.1 (64.1, 70.1)	77.3 (74.6, 79.8)	72.9 (69.9, 75.7)	58.1 (54.7, 61.3)
No	75 (73.8, 76.2)	77.5 (76.4, 78.7)	83.8 (82.8, 84.9)	81.2 (80.0, 82.3)	69.6 (68.3, 70.9)
Obesity					
Normal/underweight	73.1 (71.2, 74.9)	75.5 (73.7, 77.2)	81.8 (80.1, 83.3)	80.1 (78.4, 81.7)	67.5 (65.6, 69.4)
Overweight	74.3 (72.4, 76.1)	77.1 (75.4, 78.8)	84.0 (82.5, 85.5)	80.7 (79.0, 82.3)	69.1 (67.2, 71.0)
Obese	71.6 (69.4, 73.6)	74.5 (72.5, 76.3)	82.1 (80.3, 83.7)	78.0 (75.9, 80.0)	66.4 (64.2, 68.6)
State					
New Hampshire	71.6 (69.2, 73.9)	71.8 (69.4, 74.0)	79.6 (77.4, 81.6)	78.4 (76.1, 80.6)	65.6 (63.1, 68.0)
Oregon	73.4 (70.4, 76.2)	76.9 (74.1, 79.4)	83.5 (81.0, 85.8)	81.4 (78.6, 83.8)	69.9 (66.8, 72.8)
Washington	73.2 (71.9, 74.4)	76.0 (74.8, 77.2)	82.8 (81.7, 83.8)	79.3 (78.1, 80.5)	67.4 (66.1, 68.7)

Notes: *CI* = confidence interval. Percentages are adjusted for the following variables: gender, age, race/ethnicity, education, marital status, employment status, income, disability status, veteran status, chronic health condition, exercise, smoking, obesity, and state. Chronic health conditions include self-reported doctor-diagnosed diabetes, heart attack, angina/coronary heart disease, stroke, and asthma.

*More than high school includes adults with some college or technical school or college graduates.

**Figure 2:**
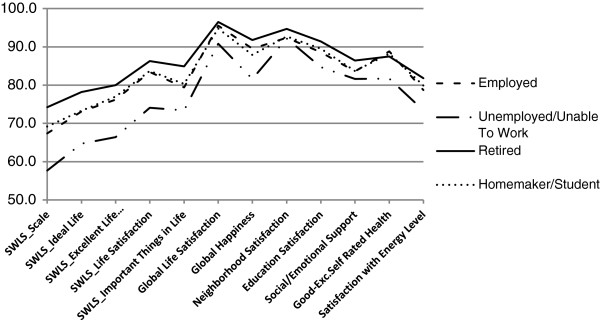
**Percent agreement with well-being domains by employment status, 2010 BRFSS pilot study, NH, OR, WA.** SWLS = Satisfaction with Life Scale [37]. For overall SWLS (“SWLS_Scale”), scores of 4 or higher, corresponding to ratings of satisfied or very satisfied, were considered positive. SWLS_Scale is based on positive responses to the four items used in this study: “In most ways, my life is close to ideal; The conditions of my life are excellent; I am satisfied with my life; So far, I have gotten the important things I want in life”.

**Figure 3:**
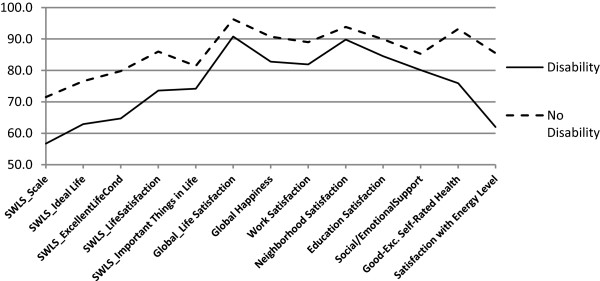
**Percent agreement with well-being domains by disability status, 2010 BRFSS pilot study, NH, OR, WA.** SWLS = Satisfaction with Life Scale [37]. For overall SWLS (“SWLS_Scale”), scores of 4 or higher, corresponding to ratings of satisfied or very satisfied, were considered positive. SWLS_Scale is based on positive responses to the four items used in this study: “In most ways, my life is close to ideal; The conditions of my life are excellent; I am satisfied with my life; So far, I have gotten the important things I want in life”.

**Figure 4:**
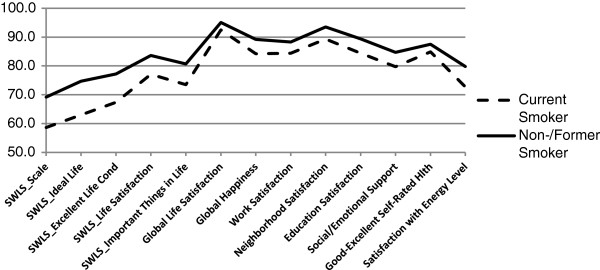
**Percent agreement with well-being domains by smoking status, 2010 BRFSS pilot study, NH, OR, WA.** SWLS = Satisfaction with Life Scale [37]. For overall SWLS (“SWLS_Scale”), scores of 4 or higher, corresponding to ratings of satisfied or very satisfied, were considered positive. SWLS_Scale is based on positive responses to the four items used in this study: “In most ways, my life is close to ideal; The conditions of my life are excellent; I am satisfied with my life; So far, I have gotten the important things I want in life”.

No differences in positive responses to the SWLS were seen between men and women. The youngest (aged 18 to 24) adults had greater life satisfaction than those between the ages of 45 and 54 years. Fewer adults aged 45 to 54 reported positive life satisfaction compared with older groups. Positive life satisfaction was more common among married adults than adults who were divorced/septed, widowed, or never married. Unemployed adults were less likely to be satisfied, and retired adults more likely to be satisfied, than employed adults (Table 2; Figure 2). Greater life satisfaction was also associated with household incomes of $75,000 or more. Adults without a disability and those who were non-smokers were more likely to report positive responses on the SWLS (Table 2; Figures 3 and 4).

In response to the global life satisfaction item, 95% of respondents reported being satisfied or very satisfied with their lives (Table 3; Figure 1). As with the SWLS, more positive responses to the global life satisfaction item were associated with being married, having incomes of $75,000 or more, not smoking, and not being a person with a disability. Adults who reported exercising (vs. not exercising) were more likely to report being satisfied with their lives, whereas those who were unemployed/unable to work (vs. employed or retired) were less likely to report being satisfied (Figure 2).

**Table 3:** Adjusted proportions of global life satisfaction, global happiness, and domain-specific life satisfaction by demographic characteristics, chronic health condition status, select behavioral risk factors, and state—Behavioral Risk Factor Surveillance System, New Hampshire, Oregon, and Washington, 2010

**Characteristic**	**Global**		**Domain-specific**		
	**Life satisfaction**	**Happiness**	**Work**	**Neighborhood**	**Education**
	**Percent (95% CI)**	**Percent (95% CI)**	**Percent (95% CI)**	**Percent (95% CI)**	**Percent (95% CI)**
N	18,489	18,530	9,696	18,442	18,396
Overall	94.6 (93.9, 95.2)	88.6 (87.7, 89.4)	87.7 (86.6, 88.8)	92.8 (92, 93.5)	88.8 (88.0, 89.6)
Gender					
Male	94.3 (93.1, 95.3)	87.6 (86.1, 88.9)	86.6 (84.8, 88.2)	93.2 (92.0, 94.3)	88.8 (87.5, 90.1)
Female	94.9 (94.0, 95.7)	89.5 (88.4, 90.5)	89.1 (87.6, 90.4)	92.4 (91.2, 93.5)	88.8 (87.6, 89.8)
Age					
18–24	94.6 (91.1, 96.7)	90.4 (86.3, 93.4)	88.9 (82.4, 93.2)	87.4 (79.3, 92.6)	87.5 (81.1, 92.0)
25–34	93.7 (91.2, 95.6)	87.7 (84.9, 90.1)	84.6 (80.9, 87.7)	89.2 (86.3, 91.5)	86.8 (84.1, 89.1)
35–44	93.7 (92.0, 95.0)	87.4 (85.4, 89.2)	86.0 (83.6, 88.1)	91.8 (90.0, 93.3)	86.1 (84.0, 87.9)
45–54	94.2 (93.0, 95.1)	86.3 (84.6, 87.8)	87.6 (85.7, 89.2)	93.8 (92.6, 94.9)	88.9 (87.3, 90.3)
55–64	95.0 (94.2, 95.8)	88.6 (87.2, 89.8)	90.4 (88.8, 91.7)	95.6 (94.8, 96.4)	91.3 (90.0, 92.3)
65–74	96.3 (95.1, 97.2)	91.6 (90.0, 93.0)	94.4 (92.3, 96.0)	96.2 (94.9, 97.1)	92.2 (90.4, 93.7)
75 or older	96.9 (95.4, 97.9)	92.0 (89.9, 93.7)	98.3 (96.6, 99.2)	96.8 (95.2, 97.9)	92.7 (90.5, 94.4)
Race/ethnicity					
White	94.7 (94.0, 95.4)	88.6 (87.7, 89.5)	87.8 (86.6, 89.0)	92.8 (91.9, 93.6)	89.0 (88.0, 89.8)
Black	92.2 (85.1, 96.1)	82.2 (73.5, 88.5)	75.1 (63.0, 84.2)	91.4 (84.5, 95.4)	84.3 (74.0, 91.0)
Hispanic	95.1 (91.4, 97.3)	90.1 (86.4, 92.9)	85.7 (78.3, 90.9)	91.9 (87.7, 94.7)	87.7 (83.7, 90.9)
Asian/Pacific Islander	94.9 (89.8, 97.5)	90.3 (85.6, 93.5)	92.7 (86.6, 96.1)	95.0 (90.7, 97.4)	90.1 (84.2, 93.9)
American Indian/Alaskan Native	91.5 (84.3, 95.6)	86.5 (78.4, 91.9)	87.7 (75.1, 94.4)	93.4 (86.8, 96.8)	89.0 (81.6, 93.6)
Other	92.6 (84.8, 96.5)	83.5 (74.0, 90.1)	88.0 (70.9, 95.7)	95.8 (90.2, 98.2)	90.2 (81.3, 95.1)
Education					
Less than high school	96.7 (94.9, 97.9)	86.9 (83.1, 90.0)	94.1 (90.3, 96.5)	91.4 (87.8, 94.0)	69.5 (63.3, 75.1)
High school graduate	95.0 (93.7, 96.0)	88.4 (86.7, 89.8)	88.9 (86.5, 90.9)	92.7 (91.2, 94.0)	85.3 (83.4, 87.0)
More than high school	94.1 (93.2, 94.9)	88.9 (87.8, 89.9)	86.7 (85.2, 88.1)	93.0 (92.0, 93.9)	92.0 (91.1, 92.9)
Marital status					
Married/living with partner	96.1 (95.3, 96.7)	91.2 (90.3, 92.1)	88.6 (87.3, 89.9)	92.4 (91.2, 93.5)	88.6 (87.4, 89.7)
Divorced/septed	92.1 (90.0, 93.8)	82.9 (80.3, 85.2)	87.0 (83.3, 90.0)	92.2 (90.1, 93.9)	86.1 (83.6, 88.3)
Widowed	93.1 (90.1, 95.3)	84.1 (80.5, 87.1)	87.9 (82.5, 91.9)	93.1 (90.3, 95.1)	89.8 (87.2, 91.9)
Never married	93.2 (91.3, 94.6)	85.2 (82.4, 87.6)	84.1 (80.1, 87.5)	94.1 (91.5, 96.0)	91.1 (88.5, 93.2)
Employment status					
Employed	95.9 (94.8, 96.7)	90.1 (88.9, 91.1)	--	92.6 (91.6, 93.5)	89.1 (87.9, 90.1)
Unemployed/unable to Work	90.8 (88.7, 92.5)	82.1 (79.2, 84.6)	--	92.0 (89.7, 93.9)	85.2 (82.3, 87.6)
Retired	96.6 (95.6, 97.3)	91.8 (90.2, 93.2)	--	94.6 (92.8, 96.0)	91.7 (89.7, 93.3)
Homemaker/student	94.6 (91.8, 96.5)	87.8 (84.3, 90.5)	--	93.1 (90.1, 95.2)	89.6 (86.6, 91.9)
Income					
< $15,000	91.0 (87.2, 93.8)	83.2 (79.1, 86.6)	76.0 (66.7, 83.4)	90.5 (87.3, 93.0)	87.8 (84.2, 90.7)
$15,000–$19,999	90.6 (86.8, 93.4)	83.3 (79.0, 86.8)	78.1 (67.6, 85.9)	90.0 (85.6, 93.1)	84.5 (80.4, 87.8)
$20,000–$24,999	93.0 (90.2, 95.1)	85.1 (82.0, 87.7)	84.6 (78.6, 89.1)	89.4 (85.6, 92.3)	87.1 (84.3, 89.5)
$25,000–$34,999	93.4 (91.2, 95.1)	87.9 (85.4, 90.1)	83.0 (78.2, 87.0)	91.7 (88.9, 93.8)	87.2 (84.7, 89.4)
$35,000–$49,999	94.7 (92.9, 96.1)	88.3 (85.9, 90.3)	85.2 (82.0, 88.0)	92.7 (90.7, 94.4)	87.3 (85.2, 89.1)
$50,000–$74,999	96.3 (94.9, 97.3)	90.3 (88.3, 92.0)	87.1 (84.3, 89.4)	93.4 (91.5, 94.8)	89.2 (87.1, 91.0)
≥ $75,000	97.5 (96.4, 98.3)	92.5 (90.9, 93.8)	91.6 (90.1, 93.0)	94.8 (93.3, 96.0)	91.8 (89.9, 93.4)
Disability Status					
Yes	91.0 (89.2, 92.5)	83.3 (81.4, 85.1)	82.1 (79.1, 84.7)	90.1 (88.1, 91.8)	85.2 (83.3, 86.9)
No	96.5 (95.7, 97.1)	91.0 (90.0, 91.9)	89.0 (87.7, 90.1)	93.8 (92.9, 94.5)	90.2 (89.3, 91.1)
Veteran					
Yes	94.5 (91.9, 96.3)	88.9 (86.7, 90.8)	86.8 (82.9, 89.8)	92.4 (90.1, 94.3)	88.5 (86.2, 90.5)
No	94.7 (93.9, 95.3)	88.5 (87.6, 89.4)	87.8 (86.6, 88.9)	92.9 (92.0, 93.6)	88.9 (87.9, 89.7)
Chronic health condition					
Yes	94.3 (93.0, 95.4)	88.1 (86.6, 89.6)	86.5 (83.9, 88.8)	92.0 (90.3, 93.3)	89.3 (87.7, 90.6)
No	94.8 (94.0, 95.5)	88.8 (87.7, 89.7)	88.0 (86.8, 89.2)	93.1 (92.2, 94.0)	88.6 (87.6, 89.6)
Exercise in past 30 days					
Yes	95.1 (94.3, 95.8)	89.6 (88.6, 90.4)	87.8 (86.6, 89.0)	92.6 (91.7, 93.4)	89.3 (88.4, 90.2)
No	93.2 (91.9, 94.3)	84.9 (83.0, 86.7)	87.2 (84.3, 89.6)	93.6 (92.0, 94.8)	87.1 (85.1, 88.8)
Current smoker					
Yes	92.4 (90.4, 94.0)	84.6 (82.2, 86.7)	84.6 (81.2, 87.5)	89.8 (87.2, 91.9)	84.9 (82.5, 87.0)
No	95.3 (94.6, 96.0)	89.6 (88.7, 90.5)	88.3 (87.1, 89.4)	93.5 (92.7, 94.2)	89.8 (88.9, 90.7)
Obesity					
Normal/underweight	93.2 (91.8, 94.4)	88.4 (86.9, 89.7)	87.5 (85.6, 89.2)	93.1 (91.8, 94.2)	88.8 (87.4, 90.1)
Overweight	95.5 (94.7, 96.2)	89.5 (88.2, 90.7)	87.6 (85.7, 89.3)	92.5 (90.9, 93.8)	89.0 (87.4, 90.4)
Obese	95.2 (94.1, 96.1)	87.8 (86.1, 89.3)	88.1 (86.0, 90.0)	92.8 (91.4, 94.0)	88.6 (87.2, 89.9)
State					
New Hampshire	94.3 (92.8, 95.6)	86.9 (85.0, 88.6)	84.5 (82.1, 86.5)	93.7 (92.2, 95.0)	89.4 (87.7, 90.9)
Oregon	94.8 (93.0, 96.3)	87.6 (85.3, 89.6)	86.2 (82.4, 89.3)	92.3 (89.8, 94.2)	89.4 (86.9, 91.4)
Washington	94.6 (93.9, 95.2)	89.2 (88.2, 90.0)	88.9 (87.6, 90.0)	92.9 (92.0, 93.6)	88.6 (87.6, 89.4)

Notes: *CI* = confidence interval. Percentages are adjusted for the following variables: gender, age, race/ethnicity, education, marital status, employment status, income, disability status, veteran status, chronic health condition, exercise, smoking, obesity, and state. Chronic health conditions include self-reported doctor-diagnosed diabetes, heart attack, angina/coronary heart disease, stroke, and asthma. Employment status is excluded from the satisfaction with work model because this item is only applicable to participants who are employed.

Satisfaction was generally high (≥87%) across specific life domains (Figure 1), with no differences by sex (Table 3). Older adults (≥65 years) reported more satisfaction from work than did younger adults (25 to 64 years). Adults with incomes of $75,000 or more were more likely to be satisfied with their work, whereas those who were black (vs. white or Asian-Pacific Islanders) were less likely to be satisfied with their work. More adults with less than a high school degree were satisfied with work compared to those with some college or technical school and college graduates. Significantly fewer adults with a disability were satisfied with work than adults without disability (Figure 3). New Hampshire adults were less satisfied with work than Washington adults. Greater neighborhood satisfaction was reported among respondents 55 years of age and older compared to those 44 years of age and younger (Table 3). Adults with incomes of $75,000 or greater were more satisfied with their neighborhoods than adults living in households earning $24,999 or less. Adults with disabilities or who smoked reported less satisfaction with their neighborhoods (Figures 3 and 4). Satisfaction with education improved with age (55 or older vs. 25–44 years), higher levels of education, and among those with incomes of $75,000 or more, but worsened among those who were unemployed/unable to work, current smokers, and adults with disabilities.

#### Happiness

As with life satisfaction, a sizable majority (89%) reported being happy or very happy (Table 3; Figure 1). Being married, having an income of $75,000 or more, and exercising were positively related to happiness, whereas those who were unemployed/unable to work (Figure 2), adults with disabilities (Figure 3), or current smokers were less happy (Figure 4).

#### Social well-being

More than three-quarters of respondents (84%) reported usually or always having the social or emotional support they need (Table 4; Figure 1). The following groups were more likely to have adequate social or emotional support when needed: those who had more than a high school education (vs. less than a high school education), were currently married (vs. not currently married), had higher incomes, or had exercised in the past 30 days. Meanwhile, these groups were less likely to report having social or emotional support: Hispanic or Asian-Pacific Islanders (vs. white), people with disabilities (Figure 3), and current smokers (Figure 4).

**Table 4:** Adjusted proportions of positive responses to social support and physical well-being items by demographics, chronic health condition status, behavioral risk factors, and state—Behavioral Risk Factor Surveillance System, New Hampshire, Oregon, and Washington, 2010

	**Social**	**Physical**	
**Characteristic**	**Social and emotional support (Always/usually)**	**Health status (Excellent/very good/good)**	**Energy level (Very satisfied/satisfied)**
	**Percent (95% CI)**	**Percent (95% CI)**	**Percent (95% CI)**
N	18,302	18,622	18,588
Overall	83.9 (82.9, 84.8)	87.3 (86.6, 88.1)	78.9 (77.9, 79.8)
Gender			
Male	82.7 (81.1, 84.2)	87.2 (86.1, 88.2)	81.8 (80.3, 83.3)
Female	85.1 (83.7, 86.3)	87.5 (86.4, 88.4)	75.9 (74.4, 77.3)
Age			
18–24	87.6 (83.0, 91.2)	93.4 (89.1, 96.1)	82.1 (75.9, 86.9)
25–34	84.4 (81.4, 87.0)	90.3 (88.1, 92.2)	77.8 (74.4, 80.8)
35–44	82.0 (79.7, 84.1)	85.9 (83.8, 87.7)	76.1 (73.8, 78.3)
45–54	81.8 (79.9, 83.6)	86.7 (85.1, 88.1)	78.0 (76.2, 79.8)
55–64	83.9 (82.3, 85.3)	85.7 (84.4, 86.9)	80.2 (78.6, 81.6)
65–74	85.2 (83.0, 87.1)	85.9 (84.3, 87.4)	80.9 (78.8, 82.9)
75 or older	84.9 (82.1, 87.4)	85.4 (83.3, 87.2)	80.7 (77.9, 83.2)
Race/ethnicity			
White	85.1 (84.1, 86.1)	87.9 (87.1, 88.6)	78.6 (77.5, 79.6)
Black	79.6 (71.2, 86.0)	82.7 (76.1, 87.8)	76.5 (68.8, 82.8)
Hispanic	77.1 (72.0, 81.5)	81.3 (77.7, 84.5)	80.3 (75.1, 84.7)
Asian/Pacific Islander	69.4 (62.1, 75.8)	86.9 (81.7, 90.8)	84.5 (77.9, 89.5)
American Indian/Alaskan Native	80.0 (69.2, 87.7)	87.5 (81.1, 92.0)	87.0 (79.3, 92.1)
Other	77.7 (66.8, 85.8)	79.3 (73.5, 84.2)	72.9 (63.5, 80.7)
Education			
Less than high school	78.3 (73.6, 82.3)	79.7 (76.4, 82.7)	81.3 (76.9, 85.0)
High school graduate	83.2 (81.2, 84.9)	85.7 (84.1, 87.1)	78.1 (76.1, 79.9)
More than high school	84.8 (83.6, 85.9)	88.8 (88.0, 89.6)	79.0 (77.7, 80.1)
Marital status			
Married/living with partner	86.1 (84.9, 87.2)	87.0 (86.0, 87.9)	79.1 (77.9, 80.3)
Divorced/septed	78.6 (75.8, 81.2)	88.1 (86.6, 89.4)	77.9 (75.2, 80.4)
Widowed	80.0 (76.8, 82.9)	87.3 (85.5, 89.0)	77.8 (74.7, 80.6)
Never married	80.9 (77.7, 83.8)	88.0 (85.8, 90.0)	79.0 (75.6, 82.0)
Employment status			
Employed	84.0 (82.6, 85.3)	89.3 (88.1, 90.3)	79.1 (77.7, 80.4)
Unemployed/unable to work	81.4 (78.5, 84.0)	81.6 (79.6, 83.4)	73.3 (70.1, 76.2)
Retired	86.2 (84.2, 88.0)	87.8 (86.4, 89.1)	81.8 (79.8, 83.7)
Homemaker/student	83.4 (79.6, 86.7)	88.4 (85.7, 90.6)	80.2 (76.8, 83.3)
Income			
< $15,000	75.0 (69.7, 79.6)	84.0 (81.2, 86.5)	76.1 (71.8, 79.8)
$15,000–$19,999	76.2 (71.0, 80.7)	81.3 (77.8, 84.5)	69.0 (63.0, 74.4)
$20,000–$24,999	79.0 (75.0, 82.5)	83.0 (80.7, 85.1)	79.5 (76.6, 82.2)
$25,000–$34,999	79.4 (75.9, 82.5)	85.1 (82.6, 87.3)	77.5 (74.8, 80.0)
$35,000–$49,999	83.0 (80.7, 85.1)	86.8 (85.1, 88.4)	77.2 (74.5, 79.7)
$50,000–$74,999	86.1 (83.9, 88.1)	90.6 (89.2, 91.8)	79.5 (77.2, 81.6)
≥ $75,000	89.7 (88.1, 91.1)	91.1 (89.7, 92.3)	81.9 (80.1, 83.6)
Disability status			
Yes	79.8 (77.6, 81.9)	75.9 (74.0, 77.7)	62.1 (59.7, 64.5)
No	85.6 (84.5, 86.6)	93.5 (92.7, 94.2)	85.6 (84.6, 86.7)
Veteran			
Yes	82.6 (79.6, 85.3)	87.4 (85.8, 88.9)	80.2 (77.6, 82.6)
No	84.1 (83.1, 85.1)	87.3 (86.5, 88.1)	78.7 (77.6, 79.7)
Chronic health condition			
Yes	82.8 (81.0, 84.5)	81.8 (80.4, 83.2)	76.7 (74.9, 78.3)
No	84.3 (83.2, 85.4)	90.3 (89.5, 91.0)	79.8 (78.6, 80.9)
Exercise in past 30 days			
Yes	84.7 (83.7, 85.7)	88.5 (87.7, 89.3)	80.6 (79.5, 81.7)
No	80.8 (78.5, 82.9)	83.6 (81.8, 85.1)	71.8 (69.4, 74.0)
Current smoker			
Yes	79.6 (76.8, 82.2)	84.8 (82.7, 86.6)	72.8 (70.0, 75.5)
No	84.9 (83.8, 85.9)	87.9 (87.1, 88.7)	80.0 (79.0, 81.1)
Obesity			
Normal/underweight	82.7 (81.0, 84.3)	88.2 (87.0, 89.4)	81.8 (80.2, 83.3)
Overweight	85.4 (83.9, 86.7)	88.9 (87.8, 89.8)	80.7 (79.2, 82.2)
Obese	83.6 (81.8, 85.2)	85.0 (83.7, 86.3)	73.3 (71.2, 75.3)
State			
New Hampshire	81.8 (79.8, 83.8)	87.9 (86.2, 89.4)	81.5 (79.6, 83.3)
Oregon	83.8 (81.2, 86.1)	87.0 (85.3, 88.6)	79.1 (76.6, 81.4)
Washington	84.2 (83.2, 85.2)	87.4 (86.5, 88.1)	78.4 (77.3, 79.5)

Notes: *CI* = confidence interval. Percentages are adjusted for the following variables: gender, age, race/ethnicity, education, marital status, employment status, income, disability status, veteran status, chronic health condition, exercise, smoking, obesity, and state. Chronic health conditions include self-reported doctor-diagnosed diabetes, heart attack, angina/coronary heart disease, stroke, and asthma.

#### Physical well-being

On physical well-being items, 87% of respondents rated their health as good to excellent, and 79% were satisfied or very satisfied with their energy levels (Table 4; Figure 1). Adults aged 18 to 34 years (vs. ≥35 years), and those who had more education were more likely to report good to excellent health, whereas Hispanics and other minorities (vs. non-Hispanic whites) and respondents with lower incomes (<$50,000) were less likely to report good health. Men and respondents with higher incomes (≥$50,000 vs. ≤$19,999) reported more satisfaction with their energy levels. New Hampshire adults reported more satisfaction with their energy levels than Washington adults. Adults who exercised reported better health and greater satisfaction with energy levels, whereas the unemployed/unable to work (Figure 2), adults with disabilities (Figure 3), those with a chronic health condition, or those who were obese (vs. normal weight or overweight) were less likely to report good health and satisfaction with energy levels. Moreover, current smokers were less satisfied with their energy levels than former smokers and nonsmokers (Figure 4).

## Discussion

This study examined mental, physical, and social well-being in population-based samples in three states. After adjustment for confounders, well-being in mental, physical, and social domains was generally high in these three states. However, almost one-third of adults in these states were dissatisfied with their lives, and their well-being differed by age, marital status, health behaviors, chronic conditions, disability status, and smoking status. As seen when characterizing employment status, disability status, or smoking status, these measures can be used to describe well-being outcomes for particular subpopulations. This suggests the measures are useful for identifying well-being disparities and for identifying subgroups with unmet needs. These findings are consistent with those of previous studies [31] and extend the few state-based studies examining well-being [31,32].

Unemployed/unable to work adults, those not currently married, adults with disabilities, current smokers, and those with lower household incomes face low levels of mental well-being as measured by the SWLS. Moreover, domain-specific well-being varied by age, marital status, employment status, race, disability, and smoking status. These differences in satisfaction with work, neighborhood, and education are important because each of these variables reflects important social determinants of health [51] and are used as indicators of social gradients and environmental or social opportunities [52]. These findings can inform programs that seek to enhance the health and well-being for specific populations. For example, about 27% of adults in these states have a physical or mental disability, making disability a priority public health issue [53]. Healthy People 2020 includes 20 objectives related to people with disabilities; some include reducing barriers to care, increasing social participation, and improving well-being.

This study found a large gap in mental well-being, assessed with the SWLS, in adults with disabilities. This finding can be used to track improvements following interventions focused on enhancing mental well-being. Findings regarding dissatisfaction with neighborhood in adults with disabilities might prompt examination of the built or social environment for people with disabilities in these states. For example, consistent with the American with Disabilities Act standards [54], are community resources accessible for people with disabilities? Are public transportation services available and accessible? Are neighborhoods safe, and do existing social norms support people with disabilities? Can people with disabilities participate in meaningful activities in their communities? Answers to these questions could aid in designing interventions that promote well-being for people with disabilities. Domain-specific findings for other subgroups prompt similar questions. For example, are some smokers who are dissatisfied with their neighborhoods living in socially isolated, unsafe, or economically depressed neighborhoods that may prompt unhealthy coping behaviors?

The pattern of well-being by age in this study pllels research on midlife development [55-57]. Poorer midlife satisfaction, as seen in this study, could be attributed to juggling job roles, family roles, and caregiving for children and aging adults [57]. Middle-aged adults are also at increased risk of depression and suicide [58]. Identifying middle-aged adults with mental illness symptoms and very low well-being in particular domains might lead to implementation of interventions for those particularly vulnerable. Previous research has also identified black and Hispanic 2007 BRFSS respondents as reporting lower global life satisfaction than whites [41]. In this study, these disparities in both life satisfaction measures disappeared after adjusting for health status, socioeconomic status, and social well-being, suggesting that these latter factors, which are important indicators of social capital, may be driving differences in life satisfaction.

More variability existed using the SWLS than the global life satisfaction measure. In general, both measures identified similar subgroups with lower well-being levels. Besides this greater variability in the SWLS, differences at the item level (“conditions of life” vs. “satisfied with life”) may reveal important drivers of well-being in different subgroups.

Social well-being findings plleled those related to mental well-being. In general, adults who were middle-aged, had a disability, or were smokers, divorced, widowed, or never married reported lower social well-being. Having supportive relationships is one of the strongest predictors of well-being [59], and low social support has been shown to contribute to about as many deaths in the US as lung cancer [60]. These findings have implications for public health and social service programs. For example, smokers with low social support levels might be at increased relapse risk following quit attempts and might benefit from messaging strategies to improve support that validates any cessation attempts and maintenance efforts [61]. Providers that serve adults with disabilities might seek to increase social connectedness for people with disabilities by supporting telephone befriending programs [62], using social media to increase their connectedness, or increasing their participation in social activities.

Physical well-being measures encompassed self-rated health status and energy level. Health status ratings generally plleled those in groups with better or worse mental and social well-being. Those with chronic health conditions and obesity, however, reporting similar mental and social well-being, also reported significantly lower health status and energy levels. Women also reported less satisfaction with their energy level than men. Because few population-based studies have examined physical well-being in this way, future studies that extend and validate these findings are needed.

This study has several limitations. First, it was limited to data from three states that are not necessarily representative of their geographic region or the US adult population. States with lower socioeconomic status and greater income inequality may fare worse. Second, the cross-sectional nature of the study design precludes determining the temporal relationship between well-being and some of the other changeable variables. Third, these data were self-reported and subject to self-presentation biases [63] that may positively skew well-being reports. Fourth, BRFSS excludes institutionalized adults and requires functional ability to participate in the survey, omitting adults who may have lower well-being levels. Fourth, the operationalization of well-being in the mental domain focused primarily on hedonic well-being measures [64]. However, the participating states placed greater value on these selected measures for their programmatic needs. Fifth, the study was limited to data from three states, not necessarily representative of more diverse states, limiting comparisons.

## Conclusion

Well-being data can help policymakers better understand population well-being when considered with more traditional economic or social indicators by providing information not captured by these indicators. These data can help tailor interventions to specific groups and communities within these states, ensuring that programs meet people’s needs and close the gap in disparities. Well-being also reflects personally meaningful outcomes that might help galvanize efforts to improve community health. The present analysis indicates that more than half of adults in these states are faring fairly well across well-being domains. This suggests that some individuals, communities, and states have resources that confer well-being individually and collectively. Policy, system change, and environmental strategies identified in the National Prevention Strategy can be cost effective ways to improve the public’s health and well-being [5]. Similar resources have been described, but warrant broader dissemination to improve population health [65-67].

HP2020 objectives for improving population well-being may galvanize national, state, and local efforts to implement evidence-based interventions such as those identified in the 2010 National Prevention, Health Promotion and Public Health Council [5,6]. Brief psychometrically sound measures like the ones used in this study can provide important information to identify vulnerable populations, identify population strengths, assess population changes in well-being due to interventions, and provide a basis for evaluating progress toward HP2020 goals.

## Competing interest

The authors declare that they have no competing interest.

## Authors’ contributions

RK developed the original study and coordinated data collection. She developed the analytical plan, assisted in interpretation of the data and results, contributed to writing the initial draft and finalized and approved the manuscript for submission. CMB served as a statistical consultant and led the statistical analysis for the study. She developed the tables for the paper, assisted in drafting the manuscript, and approved the final manuscript for submission. ML assisted in study development and conceptual design of the analysis. She developed the initial draft of the manuscript, contributed to critical revision of the manuscript, and approved the final manuscript for submission. MMZ served as the primary statistician for the study. He assisted in the interpretation of data and results. He contributed to critical revision of the manuscript, and approved the final manuscript for submission. AMB was responsible for data collection on the Washington BRFSS. She contributed to interpretation of the findings, critical revision of the manuscript and approved the final manuscript for submission. AKLH assisted with preparing the WA dataset for analysis. She reviewed and approved the final manuscript for submission. RB was responsible for data collection on the Oregon BRFSS. She contributed to interpretation of the findings, critical revision of the manuscript and approved the final manuscript for submission. KL was responsible for NH BRFSS data. He contributed to revision of the manuscript and approved the final manuscript for submission. TH contributed to statistical analysis of the data including preparing final tables. CL contributed to critical revision of the manuscript and approved the final manuscript for submission. WT contributed to critical revision of the manuscript and approved the final manuscript for submission. WHJ contributed to critical revision of the manuscript and approved the final manuscript for submission. REL contributed to critical revision of the manuscript and approved the final manuscript for submission. All authors read and approved the final manuscript.

## Disclaimer

The findings and conclusions of this study are those of the authors and do not necessarily represent the official position of the Centers for Disease Control and Prevention.
